# Responsiveness and minimal clinically important difference for pain and disability instruments in low back pain patients

**DOI:** 10.1186/1471-2474-7-82

**Published:** 2006-10-25

**Authors:** Henrik H Lauridsen, Jan Hartvigsen, Claus Manniche, Lars Korsholm, Niels Grunnet-Nilsson

**Affiliations:** 1 Clinical Locomotion Science, Institute of Sports Science and Clinical Biomechanics, University of Southern Denmark, Odense, Denmark; 2 Nordic Institute of Chiropractic and Clinical Biomechanics, Odense, Denmark; 3 Backcenter Funen, Ringe, Denmark; 4 Department of Statistics, University of Southern Denmark, Odense, Denmark

## Abstract

**Background:**

The choice of an evaluative instrument has been hampered by the lack of head-to-head comparisons of responsiveness and the minimal clinically important difference (MCID) in subpopulations of low back pain (LBP). The objective of this study was to concurrently compare responsiveness and MCID for commonly used pain scales and functional instruments in four subpopulations of LBP patients.

**Methods:**

The Danish versions of the Oswestry Disability Index (ODI), the 23-item Roland Morris Disability Questionnaire (RMQ), the physical function and bodily pain subscales of the SF36, the Low Back Pain Rating Scale (LBPRS) and a numerical rating scale for pain (0–10) were completed by 191 patients from the primary and secondary sectors of the Danish health care system. Clinical change was estimated using a 7-point transition question and a numeric rating scale for importance. Responsiveness was operationalised using standardardised response mean (SRM), area under the receiver operating characteristic curve (ROC), and cut-point analysis. Subpopulation analyses were carried out on primary and secondary sector patients with LBP only or leg pain +/- LBP.

**Results:**

RMQ was the most responsive instrument in primary and secondary sector patients with LBP only (SRM = 0.5–1.4; ROC = 0.75–0.94) whereas ODI and RMQ showed almost similar responsiveness in primary and secondary sector patients with leg pain (ODI: SRM = 0.4–0.9; ROC = 0.76–0.89; RMQ: SRM = 0.3–0.9; ROC = 0.72–0.88). In improved patients, the RMQ was more responsive in primary and secondary sector patients and LBP only patients (SRM = 1.3–1.7) while the RMQ and ODI were equally responsive in leg pain patients (SRM = 1.3 and 1.2 respectively). All pain measures demonstrated almost equal responsiveness. The MCID increased with increasing baseline score in primary sector and LBP only patients but was only marginally affected by patient entry point and pain location. The MCID of the percentage change score remained constant for the ODI (51%) and RMQ (38%) specifically and differed in the subpopulations.

**Conclusion:**

RMQ is suitable for measuring change in LBP only patients and both ODI and RMQ are suitable for leg pain patients irrespectively of patient entry point. The MCID is baseline score dependent but only in certain subpopulations. Relative change measured using the ODI and RMQ was not affected by baseline score when patients quantified an important improvement.

## Background

As clinicians and researchers we often wish to address change in a patient's condition as a result of an intervention or to distinguish individual differences in response to treatment [1]. A prerequisite for this is measurement tools that accurately assess function and monitor change over time. Standardised self-report questionnaires provide such tools and are convenient for collecting large amounts of information on for instance pain and activity limitation. Apparently similar and well-validated back-specific questionnaires have emerged over the last decade making the choice of a proper instrument for a given situation challenging [2-5]. Criteria for instrument selection have often been based on whether a particular questionnaire is reliable and valid with respect to the patient population in question but this is changing. Many authors now advocate that the property of responsiveness, defined as the ability of an instrument to detect clinically relevant change over time, is equally or even more important in the choice of an evaluative instrument [6-11]. As a consequence, no less than 31 indices have been developed and reported in the literature making both the choice of an index and comparisons between indices confusing and difficult [12].

Several approaches to classifying clinically meaningful change (responsiveness) have been proposed based on study design and the construct of change being quantified [11-16]. One such approach is the differentiation between distribution-based and anchor-based methods, the former including those based on sample variability and measurement precision. The anchor-based methods, on the other hand, include both cross-sectional and longitudinal designs which link the instrument change to a meaningful external anchor [17]. In the longitudinal designs the concept of "minimal clinically important difference" (MCID) has been introduced in an effort to define what is the smallest meaningful change score [11,18,19]. These methods have advantages and limitations and many authors propose to use both approaches [17,19,20].

Apart from the type of responsiveness index, other factors affect the size of the responsiveness index such as type of intervention, patient population under study, and timing of data collection [17,21,22]. Therefore head-to-head comparisons of responsiveness in low back pain (LBP) specific instruments in different study settings and in different subpopulations of back pain patients are of paramount importance. A literature search revealed that head-to-head comparisons has been made for 1) a general LBP population [23-30] 2), a general LBP population in relation to baseline entry scores [8,31-33], 3) specific subpopulations of back pain patients [34-36], 4) condition-specific vs. generic/patient-specific questionnaires [37-41], 5) different external criteria (anchors) [34,42], 6) pain, disability and physical impairment indices [43], and lastly as part of an instrument validation study [44-55]. Thus, concurrent comparisons of responsiveness in subpopulations of LBP patients are warranted, and to the authors' knowledge no head-to-head responsiveness assessment of LBP only versus leg pain (defined as leg pain with or without LBP) and primary sector (PrS) patients versus secondary sector (SeS) patients have been carried out.

The purpose of this study was therefore twofold: 1) to determine and compare the responsiveness of four frequently used functional status questionnaires and three pain scales when applied to four different subpopulations of low back pain patients, and 2) to determine MCID using optimal cut-points for each instrument and its dependency on baseline entry score, pain location and patient entry point.

## Methods

The study was not reported to the local ethics committee as this is not required according to the rules and regulations of the Danish scientific ethical committee. However, the study was reported to and accepted by The Danish Data Protection Agency.

### Study Population

This study is a secondary analysis of data from a large validation study of the Oswestry Disability Index in Danish [56,57]. Patients from the primary sector (7 chiropractic practices) and secondary sector (an out-patient hospital back pain clinic) of the Danish health care system were recruited. In Denmark 1/3 of the LBP patients who contact a health care practitioner for treatment are seen by a chiropractor where they receive standard active and passive conservative care. These patients are comparable to patients seen by medical doctors and physiotherapists [58]. The patients seen in the out-patient hospital back pain clinic represent a broad range of chronic LBP patients with or without leg pain who have not responded to treatment in the primary sector. These patients received multidisciplinary evaluation and treatment. Inclusion criteria were: 1) age above 18, 2) presence of low back pain and/or leg pain, and 3) able to read and understand Danish. Patients were excluded if a pathological disorder of the spine was suspected (*e.g.*, fractures, spinal infections, malignancy or inflammatory diseases). All patients received oral and written information about the project and gave their informed consent to participate in the study.

### Design

A prospective cohort study design with follow-up at one week, eight weeks and nine weeks. At baseline, one week and eight weeks follow-up, a questionnaire booklet containing sociodemographic data, medical status and outcome measures was administered to all patients. Responders at the eight weeks follow-up received a telephone interview 3–5 days after (week nine) by a specially trained professional interviewer from the Danish National Institute of Social Research. The purpose of the telephone interview was to obtain patient ratings of improvement/deterioration and the importance of such change. A detailed description of the study design can be found elsewhere [56].

### Variables

**The Oswestry Disability Index (ODI) **version 2.1 is a self-administered questionnaire measuring "back-specific function" with reference to "today" on a 10 item scale with six response categories each. Each item scores from 0 to 5 and the score is subsequently transformed into 0–100 [2,59,60,60].

**The 23-item version of the Roland Morris Disability Questionnaire (RMQ) **was developed specifically to target LBP patients with radicular symptoms and is a modification of the original RMQ [61]. We chose the 23-item version instead of the original 24-item version [62] for two reasons: 1) the 23-item version has been cross-culturally validated in Danish whereas the 24-item version has not, and 2) the psychometric properties of the two versions have been shown to be similar [63]. Each item is scaled as yes/no (scored as 1 and 0 points respectively) with the scale ranging from 0 (no disability) to 23 (extremely severe disability).

**The Low Back Pain Rating Scale (LBPRS) **was developed to measure the dimensions of pain, disability and physical impairment for patients with LBP [64]. The pain assessment index (LBPRS_pain_) is measured on 0 to 10 numerical scales with 0 representing no pain and 10 representing worst possible pain. There were three 11-box numeric rating scales (pain now, worst and average pain in the last 2 weeks) for back pain and leg pain separately. Each response scale score is added giving a scale range of 0–60 points. The disability index (LBPRS_disability_) comprises 15 items scaled as yes = 0 points, can be a problem = 1 points, no = 2 points, giving a total score of 0–30 points.

**The SF36 **is a generic 36-item questionnaire compiled from the Rand Health Insurance Long Form Health Status Scale [65]. Of the eight dimensions, we included the physical function (SF36 (pf)) and the bodily pain (SF36 (bp)) subscales. Questions are framed over a one-week period with response scales varying from dichotomous (yes/no) to six-point verbal rating scales. Each dimension is scored on a weighted 0–100 scale and an overall score is recommended [66].

**Back and/or leg pain **was scored on an 11-box numeric rating scale (pain now) from 0 (no pain) to 10 (worst possible pain) [67,68].

**The patients' global retrospective assessment of treatment effect **(transition question) was used to assess the patients' perception of their overall change in their back condition. A 7-point Likert scale transition question (TQ) ranging from "much better" to "much worse" was used [69]. Furthermore, the importance of the change in health state experienced was measured. All patients were asked to rate the question: "How important is the change you have experienced in your back and/or leg pain since the start of the treatment?" on a 0 – 10 numeric rating scale (NRS_imp_) with "very important" and "not at all important" at the extremes.

This information was collected by telephone interviews which followed a carefully planned protocol. First, all patients were told their baseline global rating of pain severity (NRS_pain_) before answering the TQ to ensure optimal patient focus on the *change *in health rather than the *present *health state [70,71]. Second, the transition question with response options was read twice. In case the patient was uncertain of which response to choose, the interviewer determined whether the patient was either better, had not changed or worse. If the interviewer decided that the patient was better the categories for being improved was read again ("much better", "better", "a little better") and similar for patients classified as worse.

All the included disability instruments have been cross-culturally adapted and validated in Danish [56,57,64,72-75]. For a complete description of the psychometric properties of each instrument we refer the reader to relevant literature reviews [2-4,60,76-78].

### Subpopulations

Patients available at the eight weeks follow-up were divided into subpopulations after either pain location or entry point in the health care system at baseline. For pain location, we looked at patients with LBP only compared to those with leg pain and/or LBP as responsiveness has been found to depend on the type of patient population studied [34,79]. The second stratification (patient entry point) was chosen as a measure of disease severity. Back pain patients initial contact with the Danish health care system is the primary sector (general practitioners, chiropractors and physiotherapists), thus, representing mostly acute conditions (≤ 30 days of pain). Comparably, referrals to a secondary sector hospital based multidisciplinary spinal unit predominantly represents patients with more chronic conditions (> 30 days of pain) [58].

### Statistical Analyses

All scales were transformed to cover an interval ranging from 0 – 100 with a high score representing higher disability or pain. This makes instruments with different scoring intervals comparable despite the fact that they are not equivalent. The raw change score for each outcome measure was obtained by subtracting the eight weeks follow-up score from the baseline score. The percentage change score was calculated as follows: [Raw change score/baseline score]*100 [80].

Responsiveness was operationalised using two strategies; standardised response mean of the raw change scores (distribution-based method) and receiver operating characteristic (ROC) curves (anchor-based method). The standardised response mean of the raw change scores (SRM_raw_) was restricted to patients who had changed and calculated as the ratio of the mean raw change score and the standard deviation of that raw change score [17,81,82]. Confidence intervals for the SRM_raw _were estimated using 200,000 bootstrap samples with replacement [83]. To compare the SRM_raw _of the different questionnaires within each subpopulation, we first estimated the SRM_raw _using stata's regression command with group indicators and the cluster option to account for intra individual correlation between responses. The differences between SMR_raw _were examined with a non-linear Wald test [84]. The same procedure was used to test the difference between "important improvement" and "no change" groups within each subpopulation.

SRM_raw _was calculated for all instruments change scores according to where the patients were seen, pain location and whether the patients had experienced an "important improvement" or "no change" (see ROC analyses). The SRM_raw _for the "important improvement" group addresses the sensitivity to change. On the other hand, the SRM_raw _for the "no change" patients addresses the important issue of specificity to change where change without clinical relevance may occur in instrument scores.

In the second strategy we used ROC curve analyses to determine sensitivity and specificity for classifying patients as having experienced an "important improvement" or "no change" and defined "important improvement" patients from two criteria: 1) had to rate themselves as either "much better" or "better" on the TQ, and 2) had to rate the importance of the change on NRS_imp _equal to or more than 7. The "no change" patients rated themselves as either "a little better", "about the same" or "a little worse" *or *with a rating of the importance of the change less than 7. Because of the low number of patients (n = 13) reporting deterioration, a "worse" sample was not included. The ROC curve is the sensitivity plotted against 1-specificity (false-positive rate) and shows the trade-off between the true-positive successes and the false-positive errors as each of several cut-off points in the change score is assessed [7,85,86]. The area under the ROC curve (ROC_auc_) can be interpreted as the ability of an instrument to discriminate between "important improvement" and "no change" patients. An area of 0.5 is interpreted as no discriminatory accuracy and 1.0 as complete accuracy [87]. An omnibus statistical comparison of the area under the ROC curve within each subpopulation was carried out using a non-parametric approach as described by DeLong et al. [88].

The MCID was determined by an optimal cut-point analysis using both the raw (MCID) and percentage (MCID_%_) change scores. The optimal cut-off change score was identified as the cut-point with equally balanced sensitivity and specificity [89] and this was considered an expression of the MCID. First, we calculated overall MCIDs, quarter-specific MCIDs by dividing the scale range into four equally sized score groups [17,32], and MCIDs specific for pain location and patient entry point. Categories with less than 10 patients were excluded from the analysis.

Second, ODI and RMQ quarter-specific MCID_% _were graphed for each subpopulation. Third, we adjusted the dependence of the MCID on the baseline score by a weighted linear regression. As the number of patients in each baseline score strata was different the regression was weighted by the number of persons used to detect each cut-point (all patients only).

All statistical calculations were analysed using the statistical package STATA^® ^v. 9.2 SE (StataCorp) and statistical significance was accepted at the *P *< 0.05 level.

## Results

Two-hundred-and-thirty-three patients with low back pain and/or leg pain were entered at baseline. At 8-weeks follow-up the response rate was 82% leaving 191 patients for analyses (PrS = 94, SeS = 97). Age and sex distributions were similar in the two patient populations and patients from the PrS had mostly low back pain only, shorter duration of the current LBP episode and used less medication compared to SeS patients. Three out of 4 disability questionnaires demonstrated significantly higher disability in the SeS patients whereas 2 out of 3 pain measures showed no difference in pain intensity levels between the two groups (Table 1).

### Distribution-based responsiveness

The mean raw change scores and SRM_raw _for the two study samples stratified according to pain location are shown in Table 2. As expected the raw change scores for the SeS sample (chronic patients) were lower in comparison to PrS sample (acute patients). To convert the transformed raw change scores to original scale scores please refer to Table 3.

The RMQ proved to be the most responsive disability measure for patients with LBP only (both PrS and SeS samples) and this was statistically significantly different from the other disability measures in the PrS patients (*P *< 0.001). For patients with leg pain the ODI and RMQ was equally responsive in PrS patients (*P *= 0.2) as was the case between the disability measures in the SeS patients. Of the 3 pain measures, the SF36 (bp) had the highest responsiveness in all subpopulations. This was statistically significant in the LBP only subgroup for both PrS and SeS patients (*P *< 0.002).

### Anchor-based responsiveness

#### Important improvement vs. no change

The proportion of patients reporting an "important improvement" was statistically higher in PrS compared to SeS patients (77% vs. 23%, *P *< 0.001) and in patients with LBP only compared to leg pain patients (71% vs. 29%, *P *< 0.001). The "important improvement" and "no change" groups had similar baseline scores except for a significantly higher mean baseline score in the improved group for: 1) RMQ in SeS patients (*P *= 0.04), 2) LBPRS_pain _(*P *= 0.04) and NRS_pain _(*P *= 0.01) in patients with leg pain.

The mean raw change scores between the "important improvement" and "no change" groups showed a significant difference for all instruments except for SF36 (pf) in leg pain patients and LBPRS_disability _in PrS and leg pain patients (data not shown).

The SRM_raw _for patients reporting an "important improvement" and patients reporting "no change" are shown in Table 4. In general, moderate to large SRM_raw _(0.7 – 2.1) were found in the "important improvement" group regardless of entry point (PrS or SeS) and pain location. As expected this was somewhat smaller in the "no change" group (0.2 – 0.9). The RMQ showed the largest difference in SRM_raw _between the "important improvement" and "no change" groups in all subpopulations when compared to the other disability measures. For the pain measures, the SF36 (bp) demonstrated the largest difference in all subpopulations except the leg pain +/- LBP patients where it was equal to the NRS_pain_.

#### Area under the ROC curve

**Figure 1:**
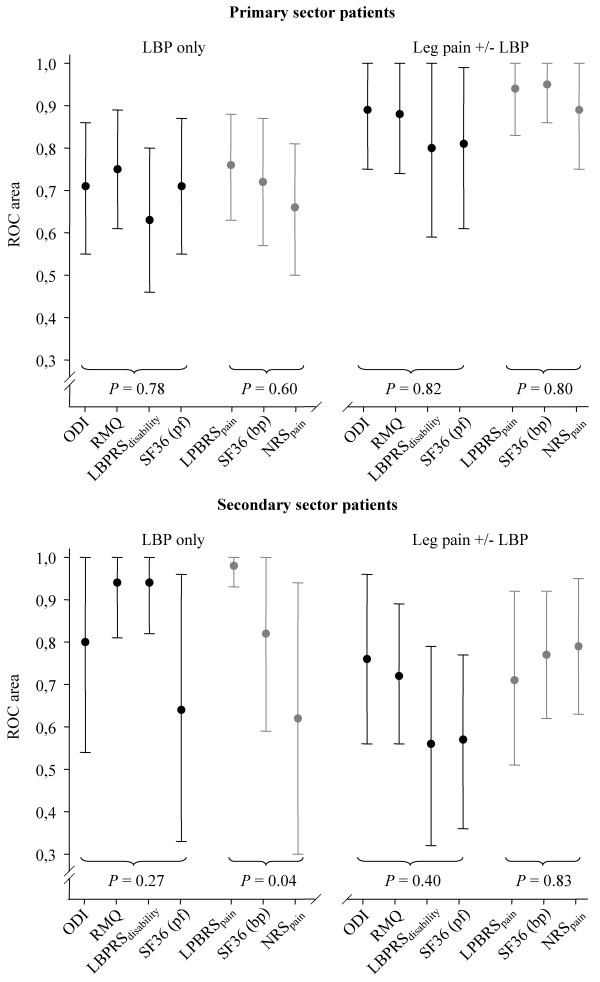
Area under the ROC curve (with 95% confidence intervals) in primary and secondary sector patients according to pain location.

Figure 1 shows the ROC_auc _with 95% CIs for all included instruments. The RMQ showed superior discriminative abilities in LBP only patients (both PrS and SeS) whereas the ODI was marginally superior in the leg pain patients, again these differences were not statistically significant. For the pain measures, the LBPRS_pain _was the superior instrument in the LBP only patients and this was statistically significant in the SeS patients (*P *= 0.04). Similar discriminative abilities were observed in the other subpopulations.

#### Minimal Clinically Important Difference

The overall and baseline-specific MCIDs for PrS, SeS, LBP and leg pain patients are presented in Table 5. Only minor variations were seen for the overall MCIDs when comparing PrS and SeS patients and LBP only and leg pain patients except for the two subscales of the SF36. MCID increased with increasing baseline entry score in the PrS sample, LBP only and leg pain +/- LBP patients. On the other hand, the dependence on baseline entry score was not monotonous for all measures in the SeS and for patients with leg pain. Poor sensitivity or specificity (< 55 [34]) were seen in 10% of the cut-point calculations.

For each 25% increase in baseline entry score (original scale range), the MCID for all patients increased by: 12 points (ODI), 2 points (RMQ), 5 points (LBPRS_disability_), 18 points (SF36 (pf)), 6 points (LBPRS_pain_), 13 points (SF36 (bp)), and 1 point for the NRS_pain_.

**Figure 2:**
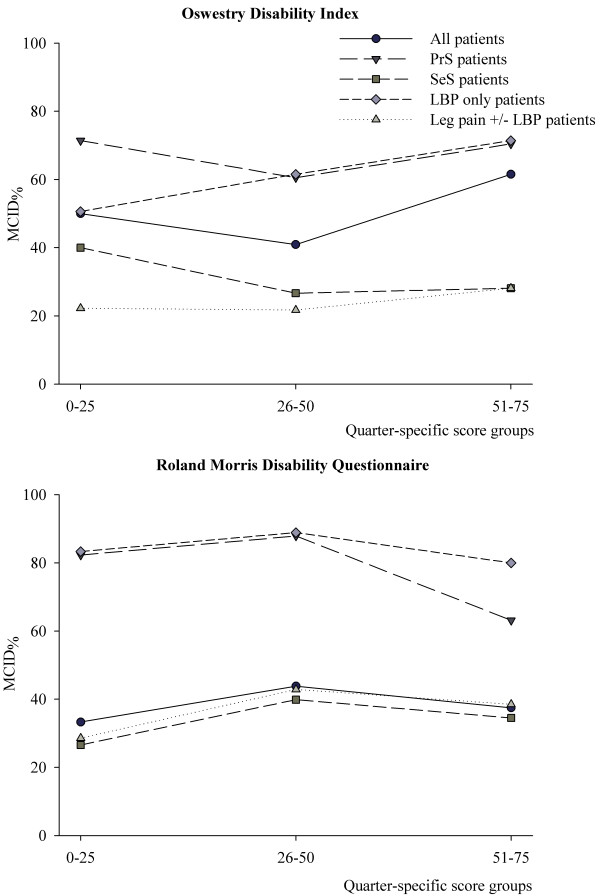
ODI and RMQ overall and quarter-specific MCIDs of the percentage change score for four LBP subpopulations.

Quarter-specific MCID_% _for ODI and RMQ are presented in figure 2. An almost constant MCID_% _across the score groups is seen for both instruments. The average MCID_% _was 51% and 38% for the ODI and RMQ, respectively. Subpopulation analyses showed that PrS and LBP patients had to change on average 65% on the ODI and 81% on the RMQ for the change to be clinically relevant. However, SeS and leg pain and/or LBP patients had to change between 28%–36% on both questionnaires – a substantially lower percentage change compared to PrS and LBP patients.

## Discussion

This is the first time a head-to-head comparison of responsiveness and MCID calculations have been carried out in 4 subpopulations of LBP patients. Furthermore, the responsiveness of the LBPRS has not been determined previously [4].

### Responsiveness

Lower change scores and SRMs were found for SeS patients. This is because the SRM is dependent on both the effectiveness of the treatment and the patient population characteristics and therefore expected to vary in a study using two distinctly different patient populations [17,21,90].

*The ODI and RMQ *have been compared in several studies, and reported SRMs for the ODI range between 0.2 and 1.9 [26,36,37,46,61,91-93] with a similar range for the RMQ (0.5–2.0) [31,41,46,61,92,93]. We found that the RMQ was most sensitive to change in patients with LBP only (significantly different in PrS patients, Table 2) whereas the ODI was slightly more responsive in leg pain patients when considering both SRM and ROC_auc_. Several authors have argued that the RMQ is more sensitive to change at lower levels of disability compared to the ODI which is sensitive to change at higher disability levels [60,91,94]. Indeed, our data showed a statistically lower mean initial disability scores in patients with LBP only compared to leg pain patients supporting this finding. Furthermore, we found the RMQ to have significantly larger differences in SRM_raw _between "important improvement" and "no change" patients in all subpopulations (Table 4).

*The LBPRS *has not been psychometrically tested for responsiveness until now, and it has been unknown how the responsiveness of this instrument compares to other functional status questionnaires [4,64]. For the disability subscale we found lower responsiveness using both SRM and ROC_auc _in comparison to the other instruments. Second, the responsiveness was conflicting depending on which strategy was used. The smaller SRMs resulted from five outliers in our dataset who showed an improvement in disability and pain on all other instruments, however, rated themselves as getting worse on the LBPRS_disability _scale. We suspect these patients have misunderstood the answer categories of the scale thus reversing a positive change score to a negative. A reanalysis omitting the outliers produced SRMs of more comparable magnitude to the rest of the disability measures. Due to the discrepancies in responsiveness according to index used and the effect of the outliers we conclude that the responsiveness of the the LBPRS_disability _is inconclusive.

*The physical function subscale of the SF36 *has been investigated in chronic LBP patients and reported SRMs range from 0.2 – 0.6 [26,37,46] and from 0.7 – 0.8 in improved patients [37,92]. It has also been suggested that the SF36 (pf) is less responsive compared to back-specific questionnaires [37,46,92]. Our results suggest that the SF36 (pf) has poorer responsiveness in patients with leg pain compared to the ODI and RMQ when considering both responsiveness indices. However, in LBP only patients the physical function scale showed lower responsiveness in SeS patients while this, remarkably, was approximately equivalent in PrS when compared to the back-specific questionnaires. Thus, we conclude that responsiveness of the SF36 (pf) is dependent on the subpopulation it is applied to.

Overall, the RMQ showed superior responsiveness and discriminative abilities in patients with LBP only which represent the more acute conditions (58% had pain ≤ 30 days) and this was irrespective of where in the health care system they were seen. However, the ODI seemed marginally superior to the RMQ in patients with leg pain +/- LBP corresponding to the more chronic conditions (66% had pain > 30 days) in both PrS and SeS patients. The LBPRS_disability _generally demonstrated lower responsiveness in comparison to the other disability measures; however, the responsiveness was conflicting according to which strategy was used.

*For the pain measures*, we found comparably higher SRMs for the SF36 (bp) in all subpopulations (range: 0.6 – 1.4) which is somewhat higher than previously published values (0.7–1.0) [26,37,92]. This finding questions the finding that the NRS_pain _is the most responsive pain scale [24,67]. However, the relatively large SRMs seen in the "no change" group signifies that some patients who indicated "no change" by the external criterion in fact changed modestly on the SF36 (bp) subscale. Reanalysing our data with a less stringent external criteria (including the "a little better" patients in the important improvement group) only altered the mean change score of the "no change" patients slightly and the SRMs remained the same (data not shown). Thus, one may question whether the specificity of the SF36 (bp) subscale is adequate when using a combined external criteria as a golden standard.

The LBPRS_pain _showed differing sensitivity to change according to which responsiveness index was used. Using SRMs the LBPRS_pain _was equally responsive to the NRS_pain_; however, using ROC_auc _it was the most responsive pain instrument in LBP only patients. Thus, we conclude that the LBPRS_pain _scale is responsive and probably preferable to the NRS_pain _as it provides more information about the pain dimension.

In summary, we found that all pain measures demonstrate similar responsiveness and this was in turn comparable to the disability measures. We recommend using the LBPRS_pain _as it is easy to use and provides more information about the patients' pain.

The optimal design and analytic strategies for a responsiveness study are topics of much debate with little or no consensus [16,95-98]. However, a recent article suggests that analytic strategies in studies of responsiveness should be based on the chosen study design and their corresponding sample change characteristics [99]. In our design we included both PrS and SeS patients to allow for subpopulation analysis and the patient composition can therefore be viewed as heterogeneous with identifiable subgroups of patients who change by different amounts. Stratford et al. argues convincingly that the proper analysis for this design would be either the area under ROC curve or Norman's S_repeat, _and our inclusion of SRMs may therefore seem obsolete. We have chosen to include both analyses as most researchers and clinicians are familiar with interpretation and application of effect sizes in comparison to ROC curves. Furthermore, the overall conclusions about responsiveness would not change (with the exception that the LBPRS_disability _subscale would have comparable responsiveness) using the ROC curve analysis alone.

### MCID, baseline entry score, entry point, and pain location

The concept of the MCID defines the smallest meaningful change score for outcome measures. An assumption behind this concept is that the instruments can indeed detect this change. Ultimately, one may question the ability of well established outcome measures to determine the smallest meaningful change as the "true" MCID is unknown. Further, the variability of the MCID is large as it is context-specific and not a fixed attribute [96].

Published MCID values for the included instruments range from: 4 – 16 points (ODI) [27,37,41,52,92,100], 3 – 5 points (RMQ) [31,32,41,60,61,63], 7 – 16 points (SF36 (pf)) [61,101], and 2–3 points for the NRS_pain _[80,102,103]. MCIDs specific to LBP patients for the SF36 (bp) and LBPRS could not be located in the literature. Our overall MCID estimates fall within reported ranges for all the instruments apart from a slightly higher MCID for the SF36 (pf). The MCIDs were generally lower for all subpopulations compared to the overall MCID, however, only minor differences were found between stratification layers (except for the subscales of the SF36). We were surprised to find similar MCIDs in the PrS and SeS samples since the perception of disease (and thus the need for improvement) has been shown to differ [58].

Stratford and Riddle have shown a large increase in MCID with increasing raw baseline score for the RMQ [32,33]. We found this pattern to be true for the overall MCID for all outcome measures and for PrS and LBP only patients (acute patients). However, using the percentage change scores of the ODI and RMQ, the MCID_% _was more or less independent of the baseline entry score for all subpopulations (figure 2). This suggests that patients relate to a percentage change in their condition rather than to an absolute change when quantifying an important improvement. Interestingly, the percentage change signifying an important improvement was dependent on the severity of the condition. PrS and LBP only patients (less severely affected) had to change significantly more (65%–81%) compared to the more severely affected SeS and leg pain +/- LBP patients (28%–36%). Maybe the more disabled leg pain patients have learned not to have too high expectations to the outcome of treatment?

Since the meaning of change varies according to baseline entry score, it seems reasonable to assume that other baseline characteristics may affect the MCID [96]. The present study examined the effect of patient entry point into the health care system (primary or secondary sector) and pain location (LBP only or leg pain) on the MCID, and found these factors to be of minor importance for most of the included disability and pain measures. An exception was the physical function and bodily pain subscales of the SF36 which showed large variations in MCID according to patient entry point and pain location.

In conclusion, we found that the overall MCID varied only slightly when stratifying patients according to point of entry into the health care system (i.e. acute vs. chronic patients) and pain location (LBP vs. leg pain +/- LBP) with the two subscales of the SF36 as an exception. Furthermore, increasing baseline entry scores resulted in greatly increased MCIDs in PrS patients and patients with LBP only. However, the dependence on baseline entry score was not monotonous for all measures in the SeS and for patients with leg pain.

### Limitations

The results of this study should be interpreted in light of several potential limitations. The classification of the ODI and RMQ as purely disability instruments may be misleading as virtually all items in each questionnaire inquire about functional activities in relation to pain [2,3,60]. Comparing these instruments to the SF36 (pf) which only measures function of daily living and to the LBPRS_disability _which partly measures pain related function (33% of the items) and function of daily living may be problematic. Second, we reported overall responsiveness and MCIDs for a broad spectrum of care-seeking LBP patients' receiving treatments ranging from simple advice to intensive multidisciplinary rehabilitation. Consequently, responsiveness and MCIDs for specific subgroups of LBP patients are likely to vary depending on such factors as entry point into the health care system, pain location, treatment received and possibly psychosocial factors, as indicated by our subgroup analyses. Statistical power issues prevented us from further sub-dividing the sample, and estimates presented are to be regarded as an overall guideline. Therefore, we recommend that researchers calculate MCIDs relevant for their individual study populations and use this when reporting the proportion of improved patients and numbers needed to treat in a clinical trial [104]. Third, the validity of using a global retrospective appraisal of change has been challenged especially with respect to recall bias [22,105]; however, this may be a minor problem [36,106]. The validity of combining two different dimensions (improvement and importance) may also be a problem since little is known about its psychometric properties. The combination was used because both improvement *and *importance is central to the concept of the MCID. Further, the cut-point used to describe who has improved or stayed the same was arbitrarily set for both dimensions. However, our results showed correlation coefficients greater than 0.63 (recommended threshold of 0.5 [105]) between the change scores and the transition question for 5 out of the 7 instruments and an expectedly lower correlation between the change scores and the rating of importance (data not shown) and between the transition question and the rating of importance (0.43). Fourth, the decision of having at least 10 patients in each baseline entry score category was arbitrarily set before the analysis was carried out. Most categories had more than 20 patients making the analysis more reliable. Lastly, some of the MCID cut-points resulted in poor sensitivity or specificity reducing the discriminative ability and validity of the cut-point. However, this occurred in only 10% of the calculations and we consider this acceptable.

## Conclusion

The RMQ appears to be more responsive mainly in patients with LBP whereas the ODI and RMQ seemed almost equally responsive in patients with leg pain irrespective of where in the health care system the patient was seen. Furthermore, the LBPRS_disability _showed inconclusive responsiveness in all subpopulations. All pain measures showed similar responsiveness with only minor differences in the subpopulations.

The MCID was only slightly affected by patient entry point and pain location whereas increasing baseline entry score increased the size of the MCIDs mainly in PrS patients and patients with LBP only. For the ODI and RMQ specifically, the percentage change score remained constant regardless of baseline score when patients quantified an important improvement. We recommend that researchers calculate MCIDs relevant for their individual study populations when reporting the results of a clinical trial.

## Competing interests

The author(s) declare that they have no competing interests.

## Authors' contributions

HHL and JH conceived the study and participated in its design and the planning of analyses. HHL drafted the manuscript, and HHL and JH revised the manuscript several times. HHL and LK made the statistical analyses. NGN and CM participated in the design of the study. All authors read and approved the final manuscript.

## Pre-publication history

The pre-publication history for this paper can be accessed here:



## Figures and Tables

**Table 1:** Baseline descriptive data for the two study populations.

	Study population	
	
Characteristic	Primary sector patients (n = 94)	Secondary sector patients (n = 97)
Age, mean (min., max.)	44 (18–76)	47 (20–85)
Sex, n (%)		
Male	44 (47)	45 (46)
Female	50 (53)	52 (54)
Pain location, n (%)		
Low back only*	70 (75)	28 (29)
Leg pain (+/- LBP)	24 (25)	69 (71)
Duration of current problem, n (%)		
≤ 30 days	69 (73)	12 (12)
> 30 days	23 (24)	79 (81)
Missing	2 (2)	6 (6)
Use of pain medication†, n (%)		
≤ a couple of times a week	59 (63)	39 (40)
> a couple of times a week	33 (35)	58 (60)
Missing	2 (2)	-
Disability at baseline 0–100 scale, mean (SD)		
ODI	27.4 (16.2)	33.7 (14.5) ‡
RMQ	40.3 (18.5)	45.2 (16.3)
LBPRS_disability_	33.6 (17.8)	45.2 (17.8) ‡
SF36 (pf)	34.9 (25.0)	45.7 (20.3) ‡
Pain at baseline, 0–100 scale (mean, SD)		
LBPRS_pain_	37.4 (21.1)	47.7 (22.5) ‡
SF36 (bp)	64.9 (20.2)	64.0 (20.5)
NRS_pain _over the past week	43.3 (23.2)	48.6 (25.3)

* The low back is defined as the area between the lower costal margin and the gluteal folds.

† Any pain-killing medication taken for back and/or leg pain over the past week.

‡ Significant different compared to primary sector patients (unpaired t-test)

**Table 2:** Mean raw Change Score (0 – 100 scale) and standardised response mean (SRM_raw_) in primary and secondary sector patients according to pain location.

Instrument	Primary Sector Patients			Secondary Sector Patients		
		
	n	Mean raw change (SD)	SRM_raw _(95% CI)	n	Mean raw change (SD)	SRM_raw _(95% CI)
**LBP only**						
ODI	69	18.5 (16.8)	1.1 (0,8; 1,4)	28	3.0 (11.7)	0.3 (-0,1; 0,6)
RMQ	69	27.7 (19.4)	1.4* (1,2; 1,7)	28	7.5 (15.7)	0.5 (0,2; 0,8)
LBPRS_disability_	67	15.4 (22.5)	0.7 (0,3; 1,0)	28	2.7 (18.6)	0.1 (-0,2; 0,5)
SF36 (pf)	70	23.6 (24.4)	1.0 (0,8; 1,2)	28	5.6 (16.9)	0.3 (0,0; 0,7)
						
LBPRS_pain_	68	18.7 (20.8)	0.9 (0,5; 1,3)	27	2.1 (16.1)	0.1 (-0,3; 0,5)
SF36 (bp)	70	40.2 (28.6)	1.4* (1,0; 1,8)	28	10.8 (17.1)	0.6* (0,2; 1,0)
NRS_pain_	69	26.2 (23.4)	1.1 (0,8; 1,5)	26	1.4 (18.1)	0.1 (-0,3; 0,5)
						
**Leg pain (+/- LBP)**						
ODI	24	12.0 (13.5)	0.9 (0,5; 1,2)	69	3.5 (9.5)	0.4 (0,1; 0,6)
RMQ	24	18.8 (22.0)	0.9 (0,5; 1,2)	68	3.6 (12.1)	0.3 (0,1; 0,5)
LBPRS_disability_	24	10.8 (17.9)	0.6 (0,2; 1,0)	69	4.5 (10.6)	0.4 (0,2; 0,7)
SF36 (pf)	24	15.5 (24.0)	0.6 (0,3; 1,0)	69	5.1 (15.4)	0.3 (0,1; 0,6)
						
LBPRS_pain_	24	23.5 (25.7)	0.9 (0,5; 1,3)	66	7.8 (15.7)	0.5 (0,2; 0,8)
SF36 (bp)	24	28.4 (26.4)	1.1 (0,6; 1,5)	69	12.3 (18.0)	0.7* (0,4; 1,0)
NRS_pain_	23	25.7 (30.4)	0.8 (0,4; 1,2)	65	7.8 (22.7)	0.3 (0,1; 0,6)

ODI = Oswestry Disability Index; RMQ = Roland Morris Disability Questionnaire; LBPRS_disability _= Disability subscale of the Low Back Pain Rating Scale; SF36 (pf) = Physical function subscale of the SF36; LBPRS_pain _= Pain subscale of the Low Back Pain Rating Scale; SF36 (bp) = Bodily pain subscale of the SF36; NRS_pain _= Numeric 11-box pain rating scale; SRM_raw _= standardised response mean of the raw change score.

*Note: *Results are shown according to where in the health care system the patients were seen and whether or not they complained of LBP or leg pain and/or LBP. The 95% CIs for the SRM_raw _were calculated using a bootstrap technique.

* Significantly larger SRM_raw _when compared to the rest of the disability/pain instruments.

**Table 3:** Relationship between raw change scores and original scale scores.

Raw change scores (0–100 scale)	Original scale change score						
	
	ODI	RMQ	LBPRS_disability_	SF36 (pf)	LBPRS_pain_	SF36 (bp)*	NRS_pain_
10	10	2.3	3	2	6	1	1
20	20	4.6	6	4	12	2	2
30	30	6.9	9	6	18	3	3
40	40	9.2	12	8	24	4	4
50	50	11.5	15	10	30	5	5
60	60	13.8	18	12	36	6	6
70	70	16.1	21	14	42	7	7
80	80	18.4	24	16	48	8	8
90	90	20.7	27	18	54	9	9
100	100	23.0	30	20	60	10	10

* After recoding of scale items.

**Table 4:** Standardised response mean (SRM_raw_) in relation to patients global important effect and according to patient entry point (primary and secondary sector patients) and pain location.

**Instrument**	**Important improvement**		**No change**		**Difference**	*P*
				
	n	SRM_raw _(95% CI)	n	SRM_raw _(95% CI)		
**Subpopulations**						
***Primary Sector Patients***						
ODI	63	1.4 (1.2; 1.7)	23	0.8 (0.4; 1.2)	0.6	0.05
RMQ	63	1.7 (1.4; 2.0)	23	0.7 (0.3; 1.1)	1.0	
LBPRS_disability_	62	0.8 (0.4; 1.2)	22	0.5 (0.1; 0.9)	0.3	
SF36 (pf)	64	1.1 (0.9; 1.3)	23	0.4 (0.0; 0.8)	0.7	
						
LPBRS_pain_	62	1.4 (1.1; 1.6)	23	0.6 (0.1; 1.1)	0.8	0.08
SF36 (bp)	64	2.1 (1.6; 2.5)	23	0.8 (0.4; 1.3)	1.2	
NRS_pain_	62	1.5 (1.2; 1.8)	23	0.8 (0.3; 1.3)	0.7	
***Secondary Sector Patients***						
ODI	19	1.0 (0.3; 1.7)	43	0.2 (-0.1; 0.5)	0.8	0.01
RMQ	18	1.3 (0.7; 1.9)	43	0.3 (0.0; 0.6)	1.0	
LBPRS_disability_	19	0.8 (0.4; 1.3)	43	0.3 (0.0; 0.7)	0.5	
SF36 (pf)	19	0.7 (0.3; 1.1)	43	0.4 (0.1; 0.7)	0.3	
						
LPBRS_pain_	18	1.1 (0.3; 1.8)	42	0.2 (-0.1; 0.5)	0.9	0.06
SF36 (bp)	19	2.1 (1.1; 3.0)	43	0.7 (0.3; 1.0)	1.4	
NRS_pain_	18	0.9 (0.4; 1.3)	41	0.2 (-0.1; 0.5)	0.7	
***LBP only patients***						
ODI	58	1.4 (1.1; 1.6)	21	0.7 (0.3; 1.1)	0.7	0.03
RMQ	58	1.7 (1.4; 2.0)	21	0.7 (0.4; 1.0)	1.0	
LBPRS_disability_	57	0.8 (0.4; 1.3)	20	0.5 (0.2; 0.9)	0.3	
SF36 (pf)	59	1.1 (0.9; 1.3)	21	0.7 (0.3; 1.0)	0.4	
						
LPBRS_pain_	57	1.3 (1.0; 1.6)	21	0.5 (0.0; 1.0)	0.8	0.07
SF36 (bp)	59	1.9 (1.5; 2.3)	21	0.9 (0.4; 1.4)	1.2	
NRS_pain_	58	1.4 (1.1; 1.7)	21	0.7 (0.3; 1.1)	0.7	
***Leg pain (+/- LBP)***						
ODI	24	1.2 (0.6; 1.7)	45	0.3 (0.0; 0.6)	0.9	0.05
RMQ	23	1.3 (0.8; 1.7)	45	0.3 (0.0; 0.6)	1.0	
LBPRS_disability_	24	0.8 (0.3; 1.2)	45	0.3 (0.0; 0.7)	0.5	
SF36 (pf)	24	0.8 (0.4; 1.2)	45	0.3 (0.0; 0.6)	0.5	
						
LPBRS_pain_	23	1.3 (0.7; 1.9)	44	0.3 (0.0; 0.6)	1.0	0.46
SF36 (bp)	24	2.0 (1.3; 2.6)	45	0.6 (0.3; 1.0)	1.1	
NRS_pain_	22	1.3 (0.8; 1.8)	43	0.2 (-0.1; 0.5)	1.1	

ODI = Oswestry Disability Index; RMQ = Roland Morris Disability Questionnaire; LBPRS_disability _= Disability subscale of the Low Back Pain Rating Scale; SF36 (pf) = Physical function subscale of the SF36; LBPRS_pain _= Pain subscale of the Low Back Pain Rating Scale; SF36 (bp) = Bodily pain subscale of the SF36; NRS_pain _= Numeric 11-box pain rating scale; SRM_raw _= standardised response mean of the raw change score.

*Note: *Results are shown according to where in the health care system the patients were seen and pain location. The 95% CIs for the SRM_raw _were calculated using a bootstrap technique. The *P *values refer to a comparison of the difference in SRMs between patients with an "important improvement" and patients with "no change" for the disability and pain instruments within each subpopulation (linear regression model with a non-linear Wald-type test).

**Table 5:** Overall and quarter-specific MCIDs (cut-points) for four low back pain subpopulations.

**Instrument**	**Overall MCID**	**Quarter-specific MCIDs by baseline entry score§**			
		
		0–25	26–50	51–75	76–100
**All patients**					
ODI	11† (11) ‡	8† (8) ‡	12† (12) ‡	40† (40)‡	
RMQ	5 (20)	2 (7)	5 (20)	6 (27)	-
LBPRS_disability_	5 (17)	1 (3)	4 (13)	10 (33)	-
SF36 (pf)	3 (17)	1 (5)	3 (17)	7 (33)	-
					
LBPRS_pain_	6 (10)	4 (7)	10 (17)	23 (38)	11† (18) ‡
SF36 (bp)	3 (29)	-	2 (22)	3 (33)	5 (50)
NRS_pain_	1 (10)	1 (10)	2 (20)	4 (40)	3 (30)

**Subpopulations**					
***Primary sector patients***					
ODI	9 (9)	9 (9)	16 (16)	40 (40)	-
RMQ	5 (20)	2 (7)*	6 (27)	11 (47)	-
LBPRS_disability_	5 (17)	0 (0)	6 (20)	10 (33)	-
SF36 (pf)	2 (11)	1 (5)*	2 (10)	9 (45)	-
					
LBPRS_pain_	12 (20)	6 (10)	13 (22)	20 (33)	-
SF36 (bp)	4 (42)	-	4 (38)*	2 (19)	5 (50)
NRS_pain_	4 (40)	1 (10)*	3 (30)	4 (40)	-
***Secondary sector patients***					
ODI	8 (8)	1 (1)*	8 (8)	18 (18)*	-
RMQ	2 (7)	4 (17)	2 (7)	2 (7)	-
LBPRS_disability_	5 (17)	1 (3)	5 (17)	3 (10)	-
SF36 (pf)	6 (30)	1 (5)	2 (10)	3 (33)*	-
					
LBPRS_pain_	8 (13)	4 (7)	10 (17)	17 (28)	-
SF36 (bp)	2 (22)	-	2 (22)*	3 (33)	3 (30)
NRS_pain_	2 (20)	1 (10)	2 (20)	4 (40)	1 (10)
***LBP only patients***					
ODI	9 (9)	9 (9)	13 (13)*	40 (40)	-
RMQ	2 (7)	2 (7)	5 (23)	11 (47)	-
LBPRS_disability_	5 (17)	1 (3)	6 (20)	13 (43)	-
SF36 (pf)	1 (5)	1 (5)	3 (17)	9 (45)	-
					
LBPRS_pain_	7 (12)	7 (12)	13 (22)	25 (42)	-
SF36 (bp)	4 (43)	-	1 (10)*	3 (33)	5 (50)
NRS_pain_	1 (10)	1 (10)	3 (30)*	4 (40)	5 (50)*
***Leg pain (+/- LBP)***					
ODI	8 (8)	3 (3)	8 (8)	18 (18)	-
RMQ	5 (20)	0 (0)	5 (20)	5 (20)	-
LBPRS_disability_	5 (17)	2 (7)*	4 (13)	5 (17)	-
SF36 (pf)	4 (20)	1 (6)*	2 (10)	7 (33)	-
					
LBPRS_pain_	10 (17)	6 (10)	5 (17)	22 (38)	11 (18)
SF36 (bp)	3 (30)	-	1 (11)	2 (19)	3 (30)
NRS_pain_	2 (20)	0 (0)	2 (20)	4 (40)	3 (30)

ODI = Oswestry Disability Index; RMQ = Roland Morris Disability Questionnaire; LBPRS_disability _= Disability subscale of the Low Back Pain Rating Scale; SF36 (pf) = Physical function subscale of the SF36; LBPRS_pain _= Pain subscale of the Low Back Pain Rating Scale; SF36 (bp) = Bodily pain subscale of the SF36; NRS_pain _= Numeric 11-box pain rating scale; MCID = Minimal Clinically Important Difference of the raw change score.

§ Baseline scores are divided into quarters of the original scale range.

† MCID in point changes of the original scales. Scale ranges: ODI 0–100; RMQ 0–23; LBPRS_disability _0–30; SF36 (pf) 0–20; LBPRS_pain _0–60; SF36 (bp) 0–10; NRS_pain _0–10.

‡ MCID in point changes of the transformed scales (0–100).

* Poor sensitivity or specificity (< 55)
